# Chemokine-like factor-like MARVEL transmembrane domain containing 6: Bioinformatics and experiments *in vitro* analyze in glioblastoma multiforme

**DOI:** 10.3389/fnmol.2022.1026927

**Published:** 2023-01-09

**Authors:** Haining Meng, Shaohua Li, Qingshu Li, Yuqin Wang, Guoan Wang, Yan Qu

**Affiliations:** ^1^Department of Emergency Medicine, Medical College of Qingdao University, Qingdao, Shandong, China; ^2^Department of Intensive Care Unit, Affiliated Qingdao Municipal Hospital of Qingdao University, Qingdao, Shandong, China; ^3^Department of Laboratory Medicine, The Third People’s Hospital of Qingdao, Qingdao, Shandong, China; ^4^Department of Intensive Care Unit, Qingdao Municipal Hospital, Qingdao, Shandong, China; ^5^Department of Emergency, Qingdao Municipal Hospital, Qingdao, Shandong, China; ^6^Qingdao Municipal Hospital, Qingdao, Shandong, China

**Keywords:** CMTM6, GBM, EMT, migration, piperlonguminine

## Abstract

**Introduction:**

Chemokine-like factor (CKLF)-like MARVEL transmembrane domain containing 6 (CMTM6) is a protein localized to the cell membrane and is known for its ability to co-localize with PD-L1 on the plasma membrane, prevent PD-L1 degradation, and maintain PD-L1 expression on the cell membrane. CMTM6 is highly expressed and plays an important role in various tumors such as oral squamous cell carcinoma (OSCC) and colorectal cancer (CRC), however, its role in Glioblastoma multiforme (GBM) is unclear.

**Methods:**

In this paper, to investigate the role of CMTM6 in GBM, we analyzed the expression of CMTM6 in GBM, the interaction with CMTM6 and the associated genes by bioinformatics. Importantly, we analyzed the expression of CMTM6 in GBM in relation to tumor-infiltrating lymphocytes (TILs), immunoinhibitors, immunostimulators, chemokines and chemokine receptors. We further analyzed the function of CMTM6 and performed *in vitro* experiments to verify it. Finally, the sensitivity of CMTM6 to drugs was also analyzed and the relationship between CMTM6 and the anticancer drug Piperlonguminine (PL) was verified *in vitro*.

**Results:**

The results showed that CMTM6 was highly expressed in GBM and correlated with multiple genes. Furthermore, CMTM6 is closely related to the immune microenvironment and inflammatory response in GBM. Bioinformatic analysis of CMTM6 correlated with the function of GBM, and our experiments demonstrated that CMTM6 significantly promoted the migration of GBM cells and epithelial-mesenchymal transition (EMT), but had no significant effect on other functions. Interestingly, we found that in GBM, PL promotes the expression of CMTM6.

**Discussion:**

In this paper, we have performed a detailed analysis and validation of the role of CMTM6 in GBM using bioinformatics analysis and in vitro experiments to demonstrate that CMTM6 may be a potential target for glioma therapy.

## 1. Introduction

Glioblastoma multiforme (GBM) is the most common primary malignancy with highly aggressive (aggressive) and devastating (devastating) nature. Despite treatment options such as surgery, chemotherapy and radiotherapy, the survival prognosis of patients remains poor and treatment options are limited (Abou-Antoun et al., 2017; Wang et al., 2019). Therefore, there is an urgent need to investigate the target genes affecting the progression of GBM to provide a theoretical basis for the treatment of GBM.

Chemokine-like factor (CKLF)-like MARVEL transmembrane domain containing 6, a type-3 transmembrane protein that was discovered as a regulator of PD-L1 (Mezzadra et al., 2017).CMTM6 is located on the cell membrane surface and can co-localize with PD-L1 recycling endosomes in the plasma membrane, increasing the PD-L1 protein half-life, preventing PD-L1 degradation, and reducing its ubiquitination to maintain PD-L1 stability in the cell membrane (Burr et al., 2017; Mezzadra et al., 2017).CMTM6, although not required for PD-L1 maturation, is specific for PD-L1, as knockdown of CMTM6 specifically reduces PD-L1 expression (Burr et al., 2017). And CMTM6 significantly enhanced the suppressive effect of PD-L1-expressing tumor cells on T cells (Mezzadra et al., 2017). This suggests that CMTM6 plays an important role in the immune response of tumors.

Chemokine-like factor (CKLF)-like MARVEL transmembrane domain containing 6 (CMTM6) is highly expressed in CRC, but the expression level is reduced in advanced tumors. The high expression of CMTM6 correlates with CD4+/CD8+ lymphocyte infiltration in CRC, demonstrating that CMTM6 has an important role in the immune microenvironment of CRC and can be a prognostic factor in CRC (Peng et al., 2021). In addition, the high expression of CMTM6 was significantly correlated with the pathological stage and macrophage infiltration of OSCC. Interestingly, the effect of CMTMT6 on macrophage polarization is that exosomes secreted by OSCC cells shuttled CMTM6 to macrophages, which in turn promoted M2-like macrophage polarization by activating ERK1/2 signaling (Pang et al., 2021). Silencing of CMTM6 in head and neck squamous cell carcinoma (HNSCC) reduces β-catenin expression, inhibits stem cell properties, suppresses epithelial-to-mesenchymal transition (EMT) and cell proliferation, and promotes CD8+ and CD4+ t-cell infiltration (Chen et al., 2020). It is suggested that CMTM6 can regulate the activity of Wnt/β-catenin signaling pathway and affect the progression of EMT and the maintenance of cancer stem cells (CSCs) as well as immune response, making it a potential target for HNSCC treatment. Knockdown of CMTM6 in hepatocellular carcinoma (HCC) cells also inhibits cell proliferation and affects tumor recurrence (Muranushi et al., 2021). In HCC patients, high expression of CMTM6 significantly shortened OS compared to patients with low CMTM6 expression (Liu et al., 2021). In addition, high CMTM6 expression is associated with poor prognosis in gastric cancer (Zhang et al., 2021) and with better prognosis in non-small cell lung cancer (NSCLC)(Hou et al., 2020). Interestingly, CMTM6 was highly expressed in OSCC chemo-naive patients and proteomic analysis of OSCC cisplatin-resistant cells revealed that CMTM6 was also significantly highly expressed. Knockdown of CMTM6 in cisplatin-resistant OSCC cell lines restored cisplatin-mediated cell death (Mohapatra et al., 2021). The above demonstrates that CMTM6 plays an important role in immune infiltration, immune response, tumor development, and oncologic drug therapy in a variety of tumors. However, the role of CMTM6A in glioma is unclear.

In this paper, we analyzed the expression of CMTM6 in pan-cancer and glioma, its interactions and associated factors by bioinformatics. We also analyzed the relationship between CMTM6 and the immune microenvironment of GBM, including the correlation with immune cells, and immune molecules. Importantly, to understand the role of CMTM6 in GBM, we analyzed its function and performed *in vitro* experiments to validate it. Furthermore, we analyzed CMTM6 by bioinformatics in correlation with the sensitivity of several drugs, demonstrating the involvement of CMTM6 in the antitumor effects of drugs. We selected a drug (piperlonguminine, PL) with unclear relationship with CMTM6 but inhibiting GBM growth for *in vitro* experiments (Liu et al., 2013, 2014), which demonstrated the relationship with CMTM6. This study demonstrates the importance of CMTM6 as a potential research target for GBM.

## 2. Materials and methods

### 2.1. Bioinformatics

ULCAN^1^ analysis of differential expression of CMTM family proteins and analysis of factors associated with CMTM6, followed by enrichment analysis and interaction network mapping using the Metascape^2^ website. GEPIA^3^ analysis of differential expression of CMTM6 in tumors. GSCA^4^ analysis of CMTM6 expression in relation to subtypes and pathway activity, mutation and drug sensitivity. GeneMANIA^5^ analysis of gene networks interacting with CMTM6. TISIDB^6^ analyzed the correlation of CMTM6 expression with lymphocyte, immunoinhibitor, immunostimulator, chemokine and chemokine receptor. CancerSEA^7^ analyzed the functional role of CMTM6 in GBM. CIBERSORT calculated the immunescore. Supplementary Figure S1 is the flow chart of this study.

### 2.2. Cell cultures

U87 and U251 cells were obtained from Shanghai Genechem. The cells were cultured with 10% fetal bovine serum (China, TransGen Biotech, FS301-02) and 1% penicillin–streptomycin liquid (China, Solarbio, P1400) of Dulbecco’s Modified Eagle’s Medium (DMEM) in a 37°C cell culture incubator (incubator) with 5% CO2.

### 2.3. Transfection

CMTM6 overexpression and knockdown lentiviral vectors (China, Genechem) with GFP tags were constructed. U87 and U251 cells were inoculated in 6-well plates and allowed to grow to 50%, lentivirally transfected with serum-free DMEM at MOI = 20 for 10 h and then changed to normal medium for further incubation. 3 days later, the cells were screened with puromycin at a concentration of 2 ug/ml (China, HANBIO, 20210412) for screening and subsequent experiments.

### 2.4. Cell counting kit-8 (CCK8)

U87 and U251 cells were seeded in 96-well plates with 7,000 cells per well, and CCK8 (United States, MedChem Express, HY-K0301) reagent was added at 24, 48, and 72 h, respectively, and then the OD value was detected by a microplate reader.

### 2.5. Wound healing

The cells were seeded in a 6-well plate with 200,000 cells per well, and a wound was scratched in each well, photographed by fluorescence microscopy at 24 h, and then the wound healing rate was calculated.

### 2.6. Transwell

Matrigel (United States, Corning, 356,234) was placed in the upper chamber of a Transwell chamber (United States, Corning, 21,721,033), placed at 37° C for 4 h, and then the liquid in the upper chamber was aspirated. Add 500 μl of medium containing 15% fetal bovine serum to the lower chamber and 200 μl of serum-free medium containing 50,000 cells to the upper chamber. Placed in a 37°C cell incubator with 5% carbon dioxide. After 24 h, they were taken out, fixed in methanol for 10 min, stained with 0.2% crystal violet (China, Solarbio, G1062) for 20 min, washed with PBS and photographed.

### 2.7. Western blot

Total cell proteins were extracted using a 99:1 mixture of cell lysate and protease inhibitor, added to loading buffer and then boiled in boiling water for 5 min. Denatured protein samples were electrophoresed on 10% SDS/polyacrylamide gel (SDS/PAGE), and then the proteins on the gel were transferred to PVDF membrane. The PVDF membranes were closed with 5% milk powder and incubated with primary antibodies for N-cadherin (United Kingdom, Abcam,ab125219,1:500), vimentin (United Kingdom, Abcam, ab125219, 1:500) and β-actin (China, Bioss, AH11286402), followed by incubation with secondary antibody (China, absin, abs20040ss, 1:5,000). Finally, exposure development was performed using an enhanced chemiluminescence kit (ECL; China, Meilunbio, MA0186).

### 2.8. Apoptosis

Cells were digested with EDTA-free trypsin, washed twice with PBS, then resuspended in binding buffer, and then APC and PI were added according to Annexin V-APC/PI Apoptosis Detection Kit (China, SIMUBIOTECH, A5001-03P-L). Incubate at room temperature for 15 min and then detect by flow cytometry.

### 2.9. Cell cycle

The cells were collected by trypsin digestion, washed twice with PBS, fixed with 70% ethanol, and placed in a 4°C refrigerator overnight. Wash twice with PBS, add appropriate amount of reagent according to PI/RNase Staining Solution Kit (China, SIMUBIOTECH, CY001-L), incubate at room temperature for 30 min in the dark, and then detect by flow cytometer.

### 2.10. Cell live–dead staining

The cells were washed twice with PBS, and then an appropriate amount of reagent was added according to the instructions of the calcein-AM/(propidium iodide)PI kit (China, Solarbio, CA1630), incubated at room temperature for 15 min in the dark, and then photographed under a fluorescence microscope.

### 2.11. Quantitative real time polymerase chain reaction

Total cellular RNA was extracted with Trizol (China, Servicebio, G3013), then DNA was removed from the total RNA using reagents (China, Accurate Biology, AG11728), followed by reverse transcription of RNA (China, Accurate Biology, AG11728) into cDNA, and finally quantitative detection was performed with FastStart Essential DNA Green Master (Germany, Roche, 58,480,300). The following is the primer sequence to detect CMTM6: Forward primer: 5′ TCACTTGGTAATTCTGGGAGC 3′; Reverse primer: 5′ GTGTACAGCCCCACTACGGA 3′.

## 3. Results

### 3.1. Expression of CMTM family In GBM, CMTM6 gene alteration and relationship with subtypes

**Figure 1:**
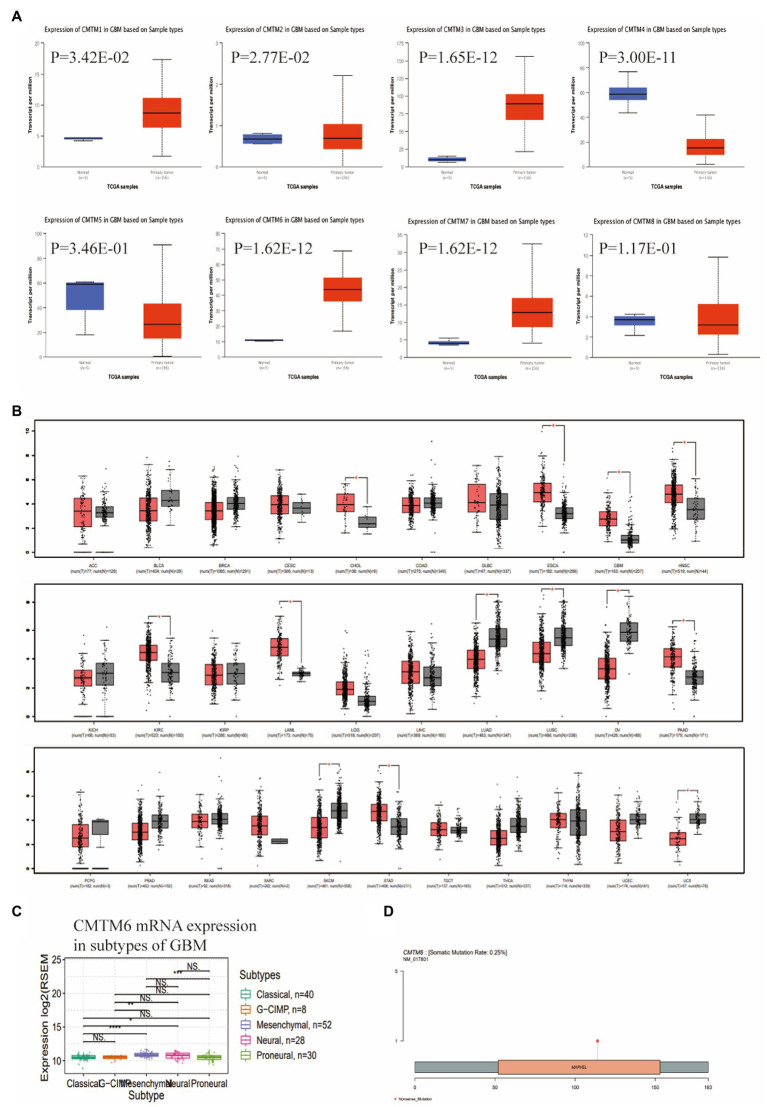
Expression of CMTM family in glioblastoma multiforme (GBM), Chemokine-like factor (CKLF)-like MARVEL transmembrane domain containing 6 (CMTM6) gene alteration and relationship with subtypes. **(A)** CMTM family expression levels in GBM were determined by UALCAN. **(B)** CMTM6 expression levels in pan-cancer from TCGA database were determined by GEPIA. **(C)** Gene alteration map of CMTM6 analyzed by GSCA website. **(D)** GSCA analysis of the relationship between CMTM6 and GBM subtypes.

We first analyzed the expression of CMTM family in GBM through the UALCAN website. The results showed that CMTM1, CMTM2, CMTM3, CMTM6 and CMTM7 were highly expressed in GBM, CMTM5 and CMTM8 were lowly expressed, while CMTM4 was not differentially expressed (Figure 1A). To further understand the potential role of CMTM6 in tumors, we further analyzed the expression of CMTM6 in pan-cancer. It showed that CMTM6 was also highly expressed in cholangio carcinoma (CHOL), esophageal carcinoma (ESCA), head and neck squamous cell carcinoma (HNSC), kidney renal clear cell carcinoma (KIRC), acute myeloid leukemia (LAML), pancreatic adenocarcinoma (PAAD), stomach adenocarcinoma (STAD), but lowly expressed in lung adenocarcinoma (LUAD), lung squamous cell carcinoma (LUSC), ovarian serous cystadenocarcinoma (OV), skin cutaneous melanoma (SKCM), uterine carcinosarcoma (UCS; Figure 1B). It indicates that CMTM6 may play different roles in different tumors. We analyzed the genetic alterations of CMTM6 through the GSCA website, showing a low mutation rate of 0.25% (Figure 1C). After that, we continued to analyze the relationship between CMTM6 and subtypes in GBM by GSCA, showing that the expression of CMTM6 was related to subtype (Figure 1D).

### 3.2. Genes associated with CMTM6 in GBM

**Figure 2:**
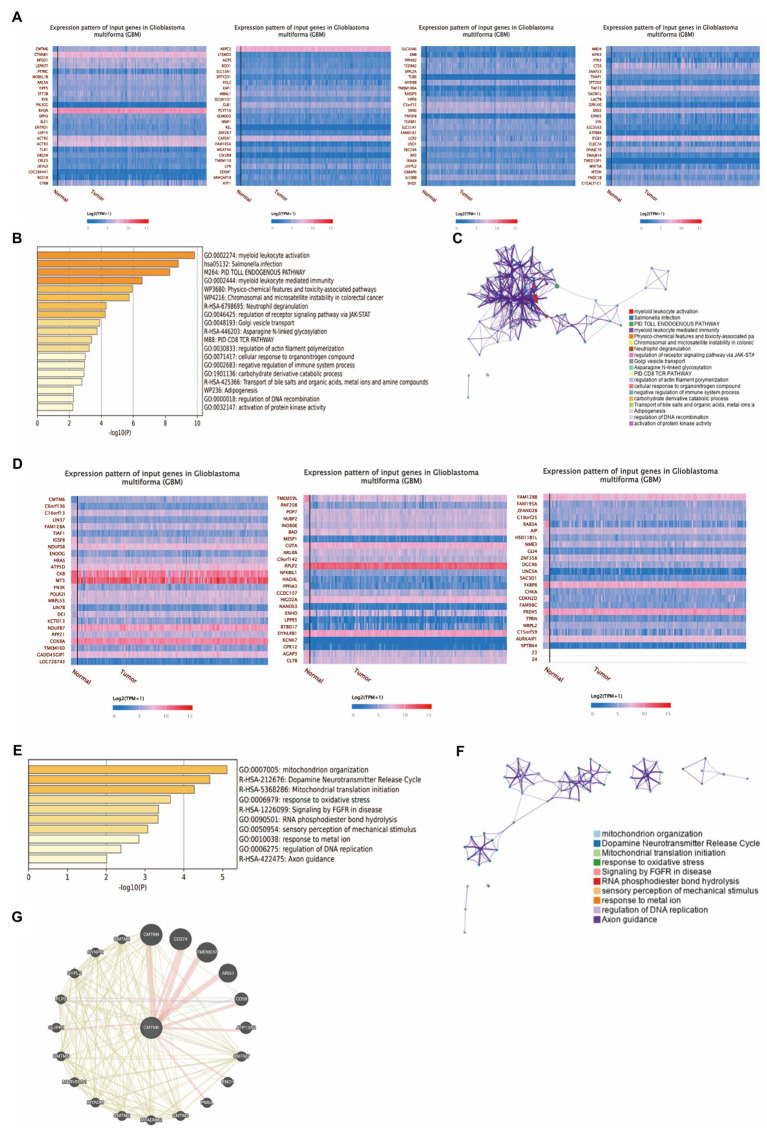
Genes associated with CMTM6 in GBM. **(A)** Heatmap of genes positively associated with CMTM6 in GBM (UALCAN). **(B)** Bar graph of enrichment of CMTM6 positively associated genes (Metascape). **(C)** Network map of CMTM6 positively associated genes (Metascape). **(D)** Heatmap of genes negatively associated with CMTM6 in GBM (UALCAN). **(E)** Bar graph of enrichment of CMTM6 negatively associated genes (Metascape). **(F)** Network map of CMTM6 negatively associated genes (Metascape). **(G)** GeneMANIA analysis of the gene network interacting with CMTM6.

To further understand the genes associated with CMTM6 in GBM, we obtained 99 genes that were positively correlated (Figure 2A) and 72 genes that were negatively correlated (Figure 2D) with CMTMT6 by UALCAN. Then we enriched 83 genes with Pearson-CC greater than or equal to 0.5 from the 99 positively associated genes in the Metascape website (Figure 2B) and network visualization analysis (Figure 2C). Similarly, 72 negatively associated genes were subjected to enrichment (Figure 2E) and network visualization analysis (Figure 2F). In addition, we analyzed the gene–gene interaction network through the GeneMANIA website and showed that there were 20 target genes interacted with CMTM6 (Figure 2G). It was demonstrated that the expression of CMTM6 in GBM was associated with multiple genes.

### 3.3. The relationship of CMTM6 with tumor-infiltrating lymphocytes (TILs), immunoinhibitors, immunostimulators and immune score

**Figure 3:**
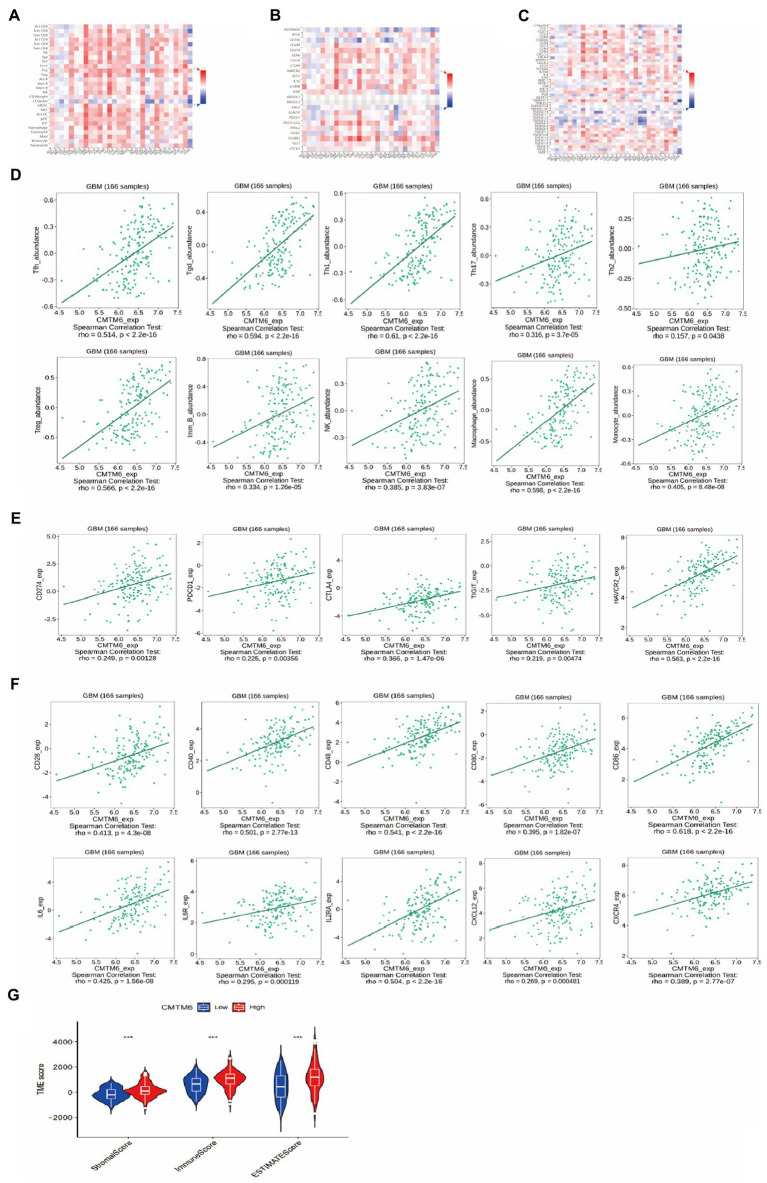
The relationship of CMTM6 with tumor-infiltrating lymphocytes (TILs), immunoinhibitors, immunostimulators and immunescore. **(A)** Heatmap of the relationship between CMTM6 and immune cells in tumors (TISIDB). **(B)** Heatmap of the relationship between CMTM6 and immunoinhibitors in pan-cancer (TISIDB). **(C)** Heatmap of the relationship between CMTM6 and immunostimulators in pan-cancer (TISIDB). **(D)** Correlation of CMTM6 with immune cells in GBM (TISIDB). **(E)** Correlation plot of CMTM6 and immunoinhibitors in GBM (TISIDB). **(F)** Correlation plot of CMTM6 with immunostimulators in GBM (TISIDB). **(G)** The relationship between CMTM6 and immunescore in GBM (CIBERSORT).

Chemokine-like factor (CKLF)-like MARVEL transmembrane domain containing 6 can stabilize PD-L1 expression, which in turn affects immune cells (Mezzadra et al., 2017). To understand the relationship between CMTM6 and immune cells, we analyzed the relationship between CMTM6 and TILs in a variety of tumors through the TISIDB website (Figure 3A). We then further analyzed the relationship between CMTM6 and TILs in GBM. The results showed that CMTM6 showed a significant positive correlation with various immune cells such as lymphocytes, macrophages and neutrophils (Figure 3D). It indicates that CMTM6 is closely related to the immune infiltration of GBM cells and may play an important role in the immune cell response to GBM.

Next, we analyzed the relationship between CMTM6 and immunoinhibitors in pan-cancer, and showed that CMTM6 was correlated with 24 immunoinhibitors in tumors (Figure 3B). To clarify the relationship between CMTM6 and each immunoinhibitor in GBM, further analysis was performed. It was shown that 17 of these 24 immunoinhibitors in GBM were correlated with CMTM6. Among them, all of them showed a significant positive correlation except CD160, which was negatively correlated with CMTM6 (Figure 3E). The above results demonstrate that CMTM6 expression is associated with a variety of immunoinhibitors and may play a role in the immunosuppressive regulation of GBM.

We have already analyzed the relationship between CMTM6 and immunoinhibitors, and then we analyze the relationship between CMTM6 and immunostimulators. First, we analyzed the relationship of CMTM6 with 45 immunostimulators in pan-cancer at the TISIDB website (Figure 3C). Similarly, to understand the correlation of CMTM6 with immunostimulators in GBM, we analyzed CMTM6 with each immunostimulator one by one. It was shown that 29 of these 45 immunostimulators were correlated with CMTM6. Among them, LTA and TNFRSF13C were negatively correlated with CMTM6, and 27 immunostimulators such as CD27, CD28 and CD40 were significantly positively correlated with CMTM6 (Figure 3F). In GBM, the expression of CMTM6 correlated with a variety of immunostimulators and immunoinhibitors, demonstrating that CMTM6 may be an important molecule affecting the immune response in GMB. Finally, we analyzed CMTM6 and immune score, which also demonstrated a close correlation between CMTM6 and immunity (Figure 3G).

### 3.4. The relationship of CMTM6 with chemokine and chemokine receptor

**Figure 4:**
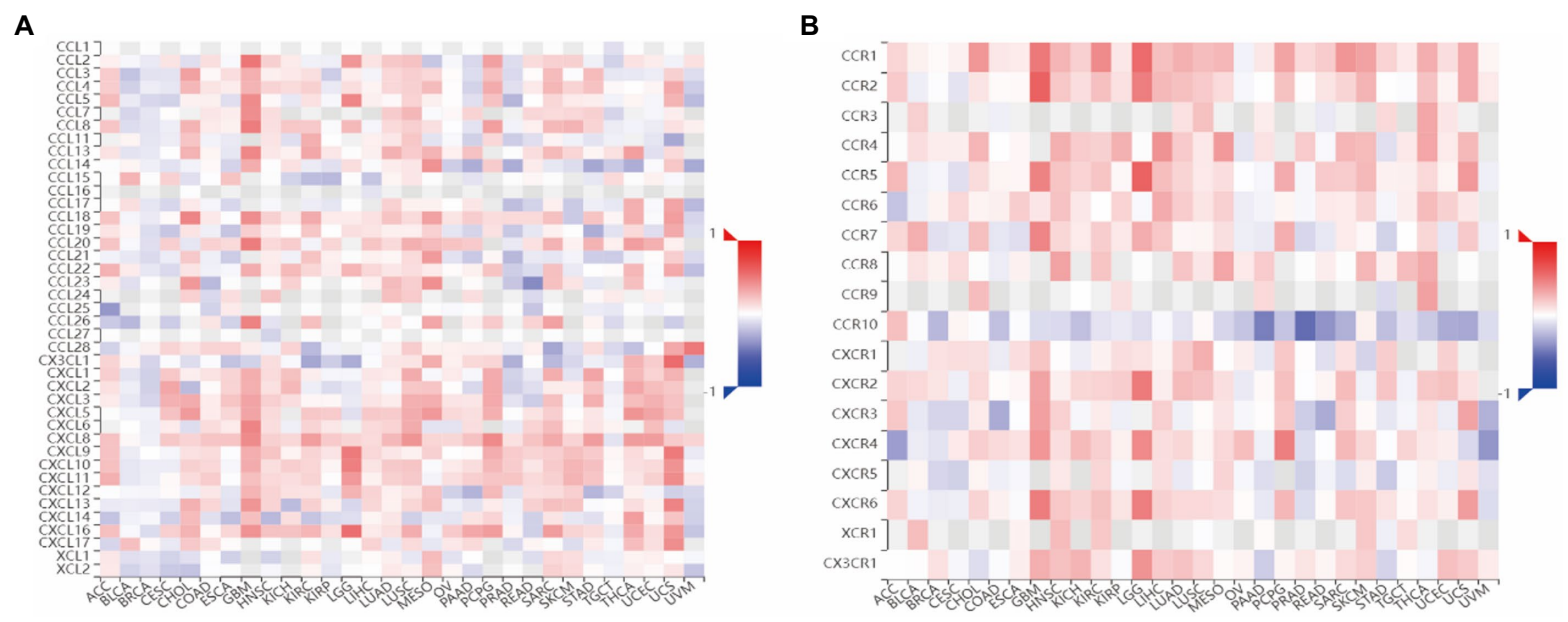
Heatmap of the relationship between CMTM6 and Chemokine, Chemokine Receptor. **(A)** Heatmap of the relationship between CMTM6 and Chemokine (TISIDB). **(B)** Heatmap of the relationship between CMTM6 and Chemokine Receptor (TISIDB).

After that, to understand the relationship between CMTM6 and chemokine and chemokine receptor, we continued our analysis through the TISIDB website. We first analyzed the relationship between CMTM6 and chemokine (Figure 4A) and chemokine receptor (Figure 4B) in pan-cancer. Further analysis showed that 26 Chemokines were positively correlated with CMTM6 in GBM, including CCL2, CCL3, and CCL4, negatively correlated with CX3CL1, and unrelated with CCL28 (Supplementary Table S1). The chemokine receptor positively correlated with CMTM6 has 10 genes including CCR1, CCR2, CXCR1, and the unrelated ones were CCR6 and CCR10 (Supplementary Table S1). This indicates that the expression of CMTM6 in GBM correlates with most chemokines and chemokine receptors.

### 3.5. Correlations between the CMTM6 and functional states

**Figure 5:**
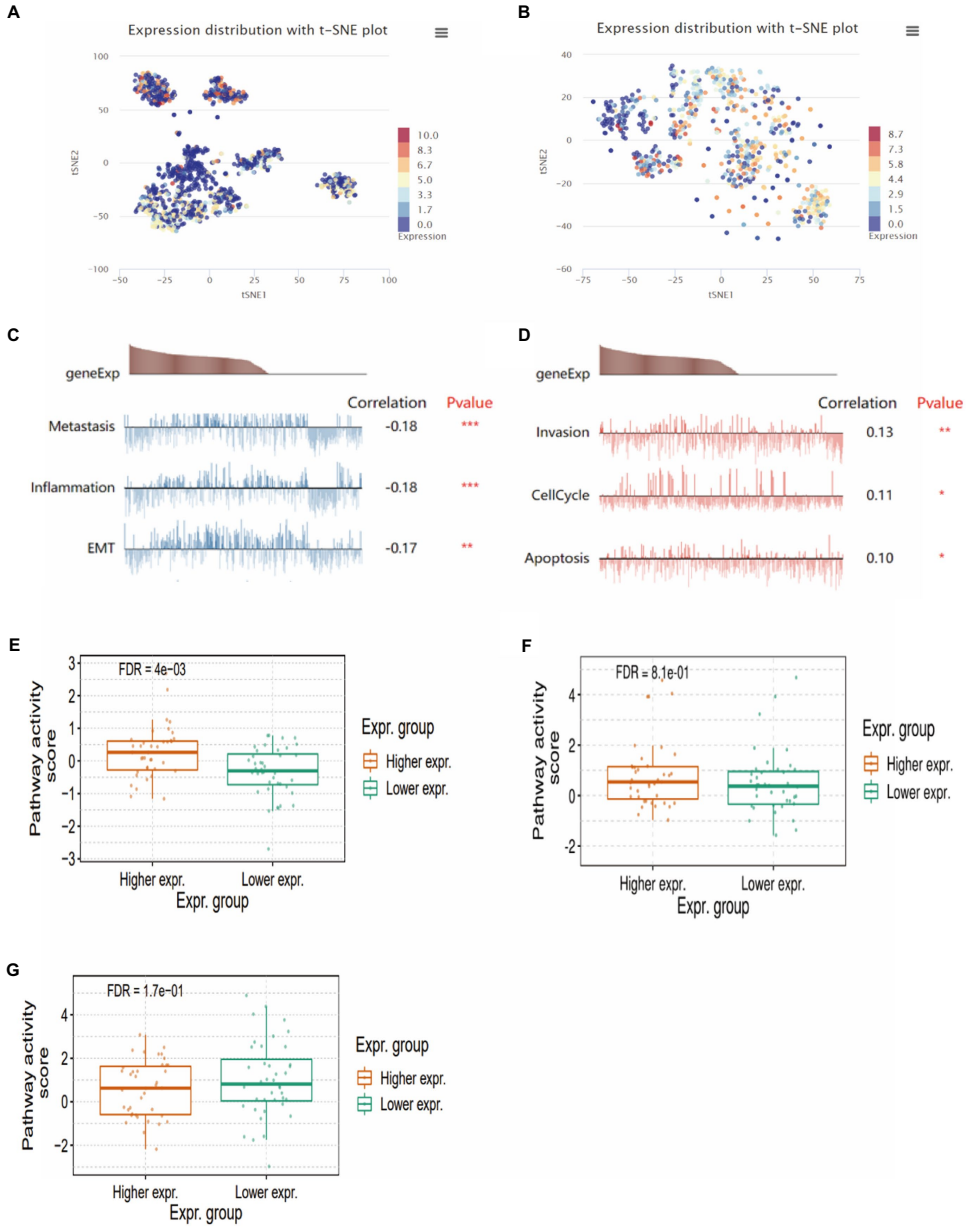
The relationship between CMTM6 and functional states. **(A,B)** CMTM6 expression clustering in GBM cell population (CancerSEA). **(C,D)** Relationship between CMTM6 expression and GMB cell function (CancerSEA). **(E)** The relationship between CMTM6 expression and EMT pathway activation (GSCA). **(F)** Relationship between CMTM6 expression and apoptosis pathway activation (GSCA). **(G)** Relationship between CMTM6 expression and cellcycle pathway activation (GSCA).

To further investigate the functional role of CMTM6 in GBM, we analyzed two single-cell datasets through the CancerSEA website and found that CMTM6 expression in GBM was heterogeneous (Figures 5A,B). Subsequently, we analyzed whether CMTM6 was associated with tumor development in two cell subsets of GBM. The results showed that cell subset 1 was correlated with metastasis, Inflammation and EMT (Figure 5C). The cell subset 2 was correlated with Invasion, cellcycle and apoptosis (Figure 5D). Of interest, we analyzed the EMT, apoptosis and cellcycle pathways in GBM and found that high expression of CMTM6 could activate the EMT pathway (Figure 5E). However, CMTM6 expression did not affect apoptosis (Figure 5F) and cellcycle pathway (Figure 5G).

### 3.6. Chemokine-like factor (CKLF)-like MARVEL transmembrane domain containing 6 (CMTM6) promotes migration and EMT of GBM cells

**Figure 6:**
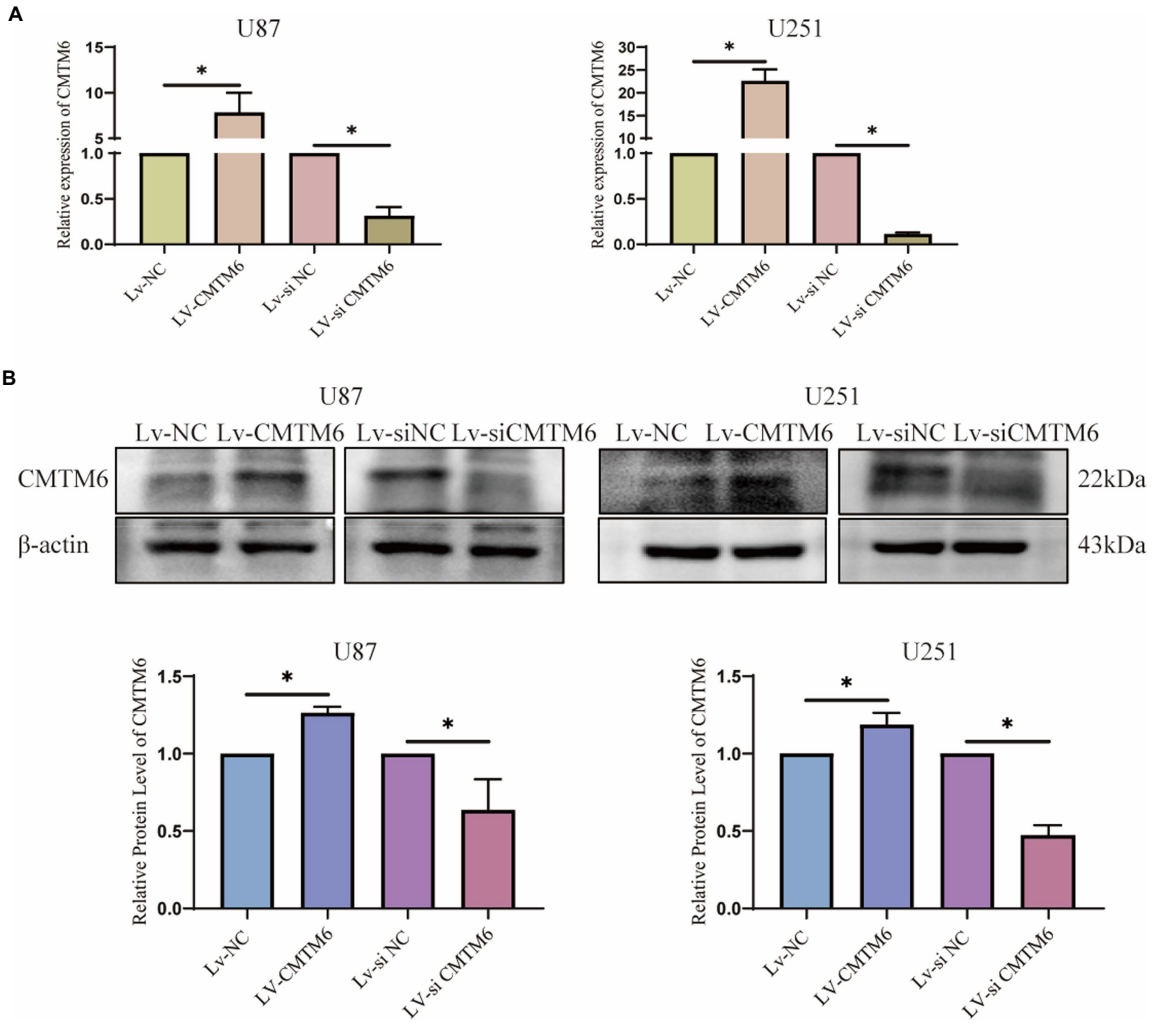
RNA and protein expression of CMTM6. **(A)** RNA level expression analysis of CMTM6 expression after up and down regulation of CMTM6. **(B)** Western blot and result analysis of CMTM6 expression after up and down regulation of CMTM6. ^*^p < 0.05.

**Figure 7:**
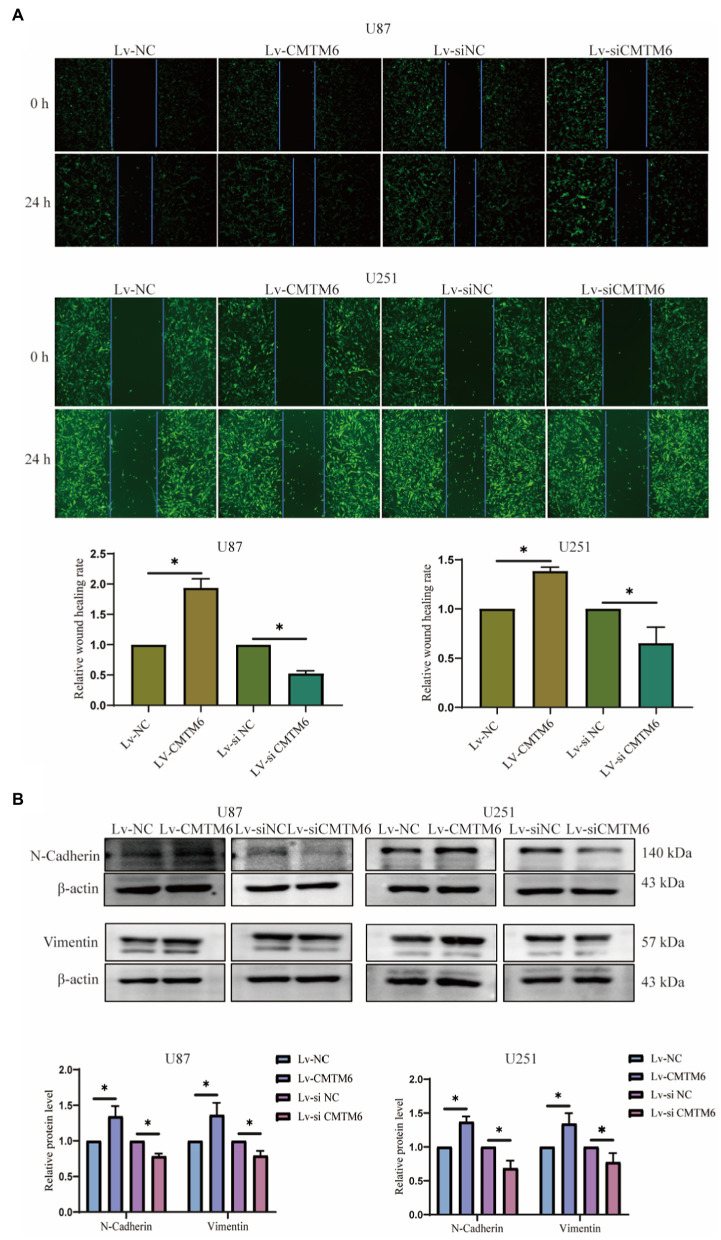
Relationship of CMTM6 with U87, U251 cell migration and EMT. **(A)** Figures of wound healing experiments and results analysis of cells after up and down regulation of CMTM6. **(B)** Western blot and result analysis of N-Cadherin and Vimentin proteins in cells after up and downregulation of CMTM6. ^*^p < 0.05.

Above, the relationship between CMTM6 and GBM cell function was analyzed, but the correlation was weak. Therefore, we performed *in vitro* experiments to verify the expression of CMTM6 and GBM cell function. First, we overexpressed and downregulated the expression of CMTM6 in U87 and U251 cells, and the results of RNA level (Figure 6A) and protein level (Figure 6B) showed that CMTM6 was successfully overexpressed and knocked down. Next, we used the wound healing to detect the changes in the migration function of cells. Compared with the control group, upregulation of CMTM6 promoted the migration of cells, while downregulation of CMTM6 inhibited the migration of cells (Figure 7A). After that, we examined the alteration of EMT, and the Western blot results showed that compared with the control group, upregulation of CMTM6 promoted the expression of N-Cadherin and Vimentin, while downregulation of CMTM6 inhibited the expression of N-Cadherin and Vimentin (Figure 7B).

### 3.7. Relationship between CMTM6 and GBM cell apoptosis, cellcycle, proliferation, and invasion

Next, we continued the experimental analysis of the functional relationship between CMTM6 and GBM cells. The results of flow cytometry showed that compared with the control group, up-regulation and down-regulation of CMTM6 had no significant effect on cell apoptosis (Supplementary Figure S2A). We also examined cell cycle changes, and again, alterations in CMTM6 expression did effect on the cellcycle (Supplementary Figure S2B). After overexpression and knockdown of CMTM6, the proliferation of cells was measured at 24, 48, and 72 h, respectively. Compared with the control group, at three time points, neither overexpression nor knockdown of CMTM6 affected cell proliferation (Supplementary Figure S3A). Finally, we examined the invasive ability of the cells by transwell assay. There was no significant change in the invasive ability of the cells after up and down regulation of CMTM6 compared to the control group (Supplementary Figure S3B).

### 3.8. Piperlonguminine promotes CMTM6 expression in GBM cells

**Figure 8:**
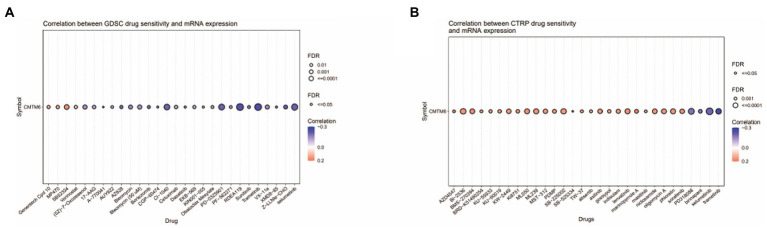
The relationship between CMTM6 and drug sensitivity in pan-cancer. (A&B) Bubble plots of top 30 drugs of genomics of drug sensitivity in cancer (GDSC) and cancer therapeutics response portal (CTRP) in relation to CMTM6 expression in pan-cancer (GSCA).

**Figure 9:**
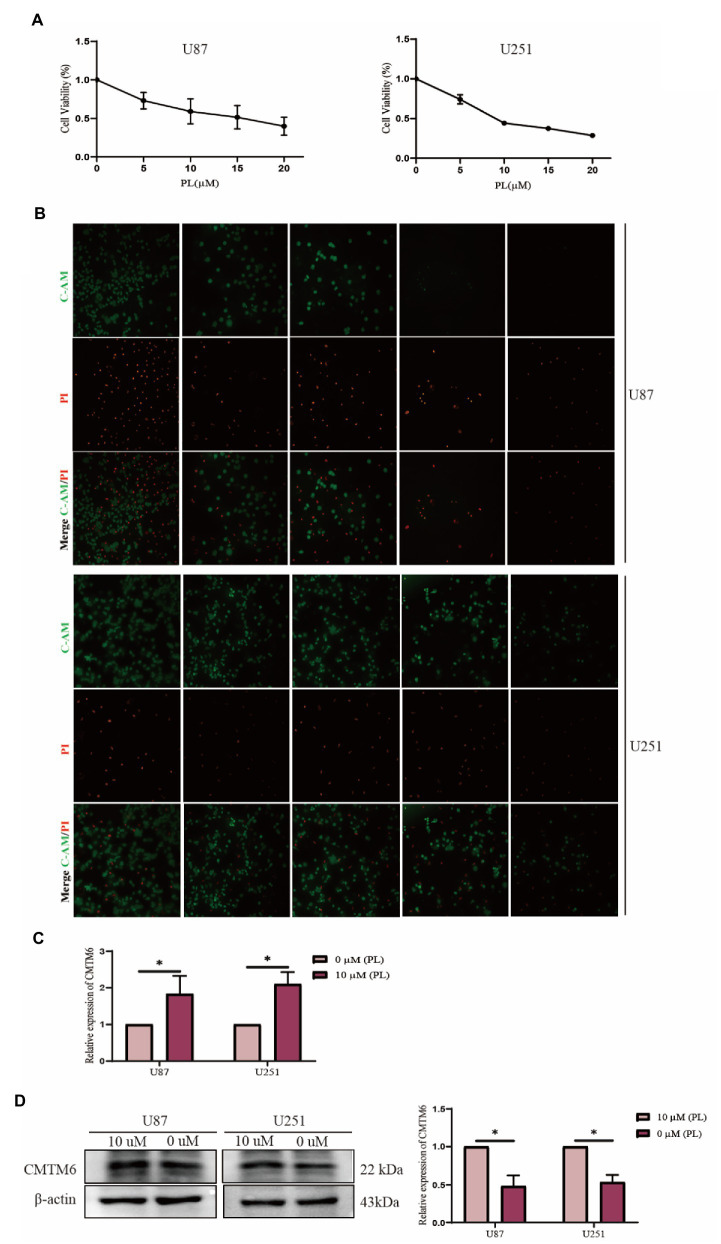
Piperlonguminine (PL) promotes the expression of CMTM6 in U87 cells. **(A)** CCK8 assay to detect the proliferation ability of U87 cells under the effect of different concentrations of PL at 0, 5, 10, 15, and 20 μM. **(B)** Live-dead cell staining assay to detect the survival of U87 cells assay under the effect of different concentrations of PL at 0, 5, 10, 15, and 20 μM. **(C)** qPCR assay to detect the change of CMTM6 mRNA level expression after 24 h of PL action on U87 cells. **(D)** Western blot plot and result analysis of CMTM6 protein levels after 24 h of PL action on U87 cells. ^*^p < 0.05.

The above analysis showed that CMTM6 was differentially expressed in various tumors (Figure 1B), so we also analyzed the relationship between the expression of CMTM6 and the sensitivity of various drugs in pan-cancer. The relationship between the top 30 drugs and CMTM6 expression in the genomics of drug sensitivity in cancer (GDSC) and the cancer therapeutics response portal (CTRP) was analyzed through the GSCA website (Figures 8A,B). The results showed that the expression of CTMT6 was associated with multiple drug sensitivity in tumors. Previous studies have shown that PL can selectively kill GBM cells and inhibit GBM cell migration (Liu et al., 2013, 2014). Interestingly, CMTM6 can also inhibit the migration of GBM cells. However, in GBM, the relationship between CMTM6 expression and PL is unclear. We first added 0, 5, 10, 15, and 20 μM of PL to U87 and U251 cells. Live-dead cell staining and CCK8 experiments showed that 10 μM could significantly inhibit cell proliferation (Figure 9A) and survival (Figure 9B). However, when PL was higher than 10 μM, the cell viability was significantly reduced (Figure 9A), so we chose 10 μM for subsequent experiments.

Piperlonguminine was applied to U87 cells for 24 h, and then the expression changes of CMTM6 were detected. The results of the RNA level assay showed that 10 μM promoted the expression of CMTM6 compared with the 0 μM group (Figure 9C). The protein level assay showed that the 10 μM group significantly promoted the expression of CMTM6 compared with the 0 μM group (Figure 9D).

## 4. Discussion

The expression of CMTM6 plays an important role in tumor progression, but its role in GMB is unclear. In this paper, the expression and related roles of CMTM6 in GBM were analyzed and verified by *in vitro* experiments. First, we analyzed the expression of the CMTM family in GMB, and the results showed that CMTM6 was significantly highly expressed in GBM. Moreover, the expression of CMTM6 in GBM was correlated with the subtype (Subtype). This demonstrates that CMTM6 may be a potential target gene for studying GBM. In order to have a clearer understanding of the role of CMTM6 expression in tumors, we also analyzed CMTM6 expression in pan-cancer and found that CMTM6 was differentially expressed in a variety of tumors. For example, CMTM6 expression was significantly higher in OSCC than in adjacent tissues (Zhang et al., 2021) and is widely expressed in melanoma, which is associated with a good prognosis (Martinez-Morilla et al., 2021). In addition, CMTM6 expression was associated with differentiation in lung cancer (Hou et al., 2020), with a better prognosis in OV (Yin et al., 2022), and significantly higher in triple-negative breast cancer (TNBC) than HER2-driven breast cancer (Tian et al., 2021). These demonstrate that CMTM6 does play a role in a variety of tumors.

We analyzed 20 genes interacting with CMTM6, among which CMTM4, CD274, TMEM237, ARG1 and CD58 showed the most significant correlation with CMTM6. Interestingly, it was shown that co-expression of CMTM6 and CMTM4 on gastric cancer mesenchymal cells and epithelial cells predicts a poor prognosis and affects the effect of PD-1/PD-L1 immunotherapy (Wang et al., 2021). Next, we screened 99 genes positively and 72 negatively correlated with CMTMT6 to understand the genes associated with CMTM6 in GBM. This indicates that CMTM6 expression is associated with multiple genes in GBM, and further confirms the large network of relationships in GBM and the potential role that CMTM6 expression may play.

Chemokine-like factor-like MARVEL transmembrane domain containing 6 acts as a stabilizer of PD-L1, and the expression of CMTM6 is closely related to the tumor microenvironment (Zhao et al., 2020; Liang et al., 2022; Yaseen et al., 2022). Among them, in head and neck squamous cell carcinoma (HNSCC), downregulation of CMTM6 not only decreases PD-L1 expression but also promotes infiltration of CD4+ and CD8+ T cells (Chen et al., 2020). In HCC, CMTM6 expression was positively correlated with T lymphocytes (CTL) infiltration (Muranushi et al., 2021). In LUAD, CMTM6 expression was positively correlated with the level of infiltration of immune cells of macrophages M1/M2, dendritic cells, neutrophils, and CD4 T cells (Wang et al., 2020). In contrast, in lung squamous carcinoma (LUSC), CMTM6 was negatively correlated with CD4 + T cells, positively correlated with CD8 + T cells, and positively correlated with dendritic cells, macrophages, and neutrophils (Shang et al., 2020). Chemokine-like factor (CKLF)-like MARVEL transmembrane domain containing 6 is also highly expressed in CRC and positively correlates with CD4 + and CD8 + TILs (Peng et al., 2021). And in clear cell renal cell carcinoma (ccRCC), CMTM6 silencing leads to increased CD4 + and CD8 + T cell infiltration in mouse models, which in turn promotes anti-tumor immunity (Wang et al., 2022). These demonstrate that CMTM6 expression affects the immune microenvironment of tumors. However, the role of CMTM6 expression in the GBM tumor microenvironment is unclear, so we analyzed the relationship between CMTM6 and GBM immune cells. The results showed that CMTM6 expression was positively correlated with immune cells such as T cells, B cells, NK cells and monocyte cells. It showed that CMTM6 expression also significantly affected the infiltration of immune cells in GBM, and the high expression may promote the infiltration of immune cells. In addition, CMTM6 expression was also closely correlated with immunoinhibitors and immunostimulators. Our analysis showed that the expression of CMTM6 correlated with 24 immunoinhibitors, of which 23 were positively correlated with PDCD1, CD274, CD244, CD96, CTLA4, HAVCR2, IL-10, except for a negative correlation with CD160. In addition, CMTM6 expression was correlated with 45 immunostimulators, of which only LTA and TNFRSF13C were negatively correlated with CMTM6, and 43 immunostimulators such as CD27, CD28, CD40, CD48, CD80, CXCL12, CXCR4 and IL-6 were positively correlated with CMTM6. Interestingly, the expression of CMTM6 also correlated with the expression of chemokine and chemokine receptor. In GBM, CMTM6 was positively correlated with 26 chemokines such as CCL2-5, CXCL1-3, CXCL8-14 and 10 chemokine receptors such as CCR1, CCR2, CXCR1, and only negatively correlated with CX3CL1. These indicate that the expression of CMTM6 in GBM is not only related to immune cell infiltration but also closely related to immune response and immune microenvironment.

We analyzed the relationship between CMTM6 expression and GBM function on CancerSEA website. CMTM6 correlated with metastasis, inflammation, EMT, invasion, cell cycle and apoptosis, but the correlation coefficient was weak. So we performed *in vitro* experiments to validate in GBM cells U87 and U251. We overexpressed CMTM6 in U87 and U251, which promoted cell migration and expression of N-cadherin and vimentin. Silencing of CMTM6 inhibited cell migration and N-cadherin, vimentin expression. However, altering CMTM6 did not affect cell proliferation, apoptosis, cellcycle and invasion. This indicates that the expression of CMTM6 only affected the migration and EMT of GBM cells in *in vitro* experiments, and was positively correlated. Correspondingly, the results of the GSCA website analysis also showed that high expression of CMTM6 could activity of EMT pathway, but not activate apoptosis and cellcycle pathway. Currently, there are also some studies on the function of CMTM6 in tumors. In HCC, the expression of CMTM6 is significantly reduced and can inhibit cell proliferation by blocking the G1/S phase of cells (Muranushi et al., 2021; Huang et al., 2022). However, some studies have also shown that the expression of CMTM6 is positively correlated with vimentin, and interacts with vimentin to promote EMT, which in turn promotes migration, proliferation and invasion, and high expression of CMTM6 predicts poor prognosis in HCC (Huang et al., 2021; Yugawa et al., 2021). In addition, CMTM6 was significantly upregulated in ccRCC, and knockdown of CMTM6 significantly reduced ccRCC cell proliferation, migration, and invasion in an *in vitro* and *in vivo* mouse model (Wang et al., 2022). All of these indicate that CMTM6 expression is associated with tumor development and may be a new target for tumor research.

Currently, studies have shown that CMTM6 is associated with tumor drug sensitivity. For example, CMTM6 sensitizes HCC cells to cisplatin and doxorubicin (Huang et al., 2022), and knockdown of CMTM6 in OSCC cells restores cisplatin-mediated cell death, and conversely overexpression rescues the drug-resistant phenotype of the cells (Mohapatra et al., 2021). So we analyzed the relationship of the top 30 drugs in GDSC and CTRP with CMTM6, showing that CMTM6 was associated with sensitivity of some drugs. It is worth noting that a drug PL inhibits the migration of GMB cells and selectively kills GBM cells, while CMTM6 also affects the migration of GBM cells, but the relationship between PL and GBM has not been reported yet. Therefore, we examined the correlation between PL and CMTM6 expression in U87 and U251 cells. The results showed that PL promoted the expression of CMTM6. But the deep mechanism of the relationship between PL and CMTM6 needs further study.

Our experiments have some limitations and further *in vivo* and *in vitro* experiments are needed to explore the relevance of CMTM6 expression to the GBM immune microenvironment, functional mechanisms and drugs.

In conclusion, our study analyzed that CMTM6 is highly expressed in GBM and is closely associated with the immune microenvironment. Importantly, high expression of CMTM6 promotes migration and EMT of GBM cells. In addition, the drug PL, which affects GBM progression, can promote the expression of CMTM6. It indicates that CMTM6 may be a new target for the study of GBM, and we hope that our study can provide a little help for the study of GBM.

## Data availability statement

The raw data supporting the conclusions of this article will be made available by the authors, without undue reservation.

## Author contributions

YQ and GW conceived and designed the study and critically reviewed the manuscript. SL, QL and YW performed literature search and analyzed the data and generated the figures and tables. HM wrote the manuscript and conducted experimental studies. All authors contributed to the article and approved the submitted version.

## Funding

The work was supported by the National Natural Science Foundation of China for funding (Fund No. 81971873).

## Conflict of interest

The authors declare that the research was conducted in the absence of any commercial or financial relationships that could be construed as a potential conflict of interest.

## Publisher’s note

All claims expressed in this article are solely those of the authors and do not necessarily represent those of their affiliated organizations, or those of the publisher, the editors and the reviewers. Any product that may be evaluated in this article, or claim that may be made by its manufacturer, is not guaranteed or endorsed by the publisher.

## Supplementary material

The Supplementary material for this article can be found online at: https://www.frontiersin.org/articles/10.3389/fnmol.2022.1026927/full#supplementary-material

Supplementary Figure S1 Flow chart of this study.

Supplementary Figure S2 The relationship between the expression of CMTM6 and the apoptosis and cellcycle of cells. **(A)** Flow cytometric apoptosis and result analysis of cells after up and down regulation of CMTM6. **(B)** Flow cellcycle and results analysis of cells after up and down regulation of CMTM6. *p<0.05.

Supplementary Figure S3 The relationship between the expression of CMTM6 and the proliferation and invasion of cells. **(A)** Changes in cell proliferation ability at 24 h, 48 h and 72 h after up and down regulation of CMTM6. **(B)** Transwell invasion and result analysis of cells after up and down regulation of CMTM6. *p<0.05.

## Footnotes

^1^ http://ualcan.path.uab.edu/index.html

^2^ https://metascape.org/gp/index.html#/main/step1

^3^ http://gepia.cancer-pku.cn/detail.php

^4^ http://bioinfo.life.hust.edu.cn/GSCA/#/

^5^ https://genemania.org/

^6^ http://cis.hku.hk/TISIDB/index.php

^7^ http://biocc.hrbmu.edu.cn/CancerSEA/
