# Supplementary figures and images for: Chemokine-like factor-like MARVEL transmembrane domain containing 6: Bioinformatics and experiments in vitro analyze in glioblastoma multiforme
Source: Front Mol Neurosci. 2023 Jan 9;15:1026927. doi: 10.3389/fnmol.2022.1026927 (PMC9869805; doi:10.3389/fnmol.2022.1026927)

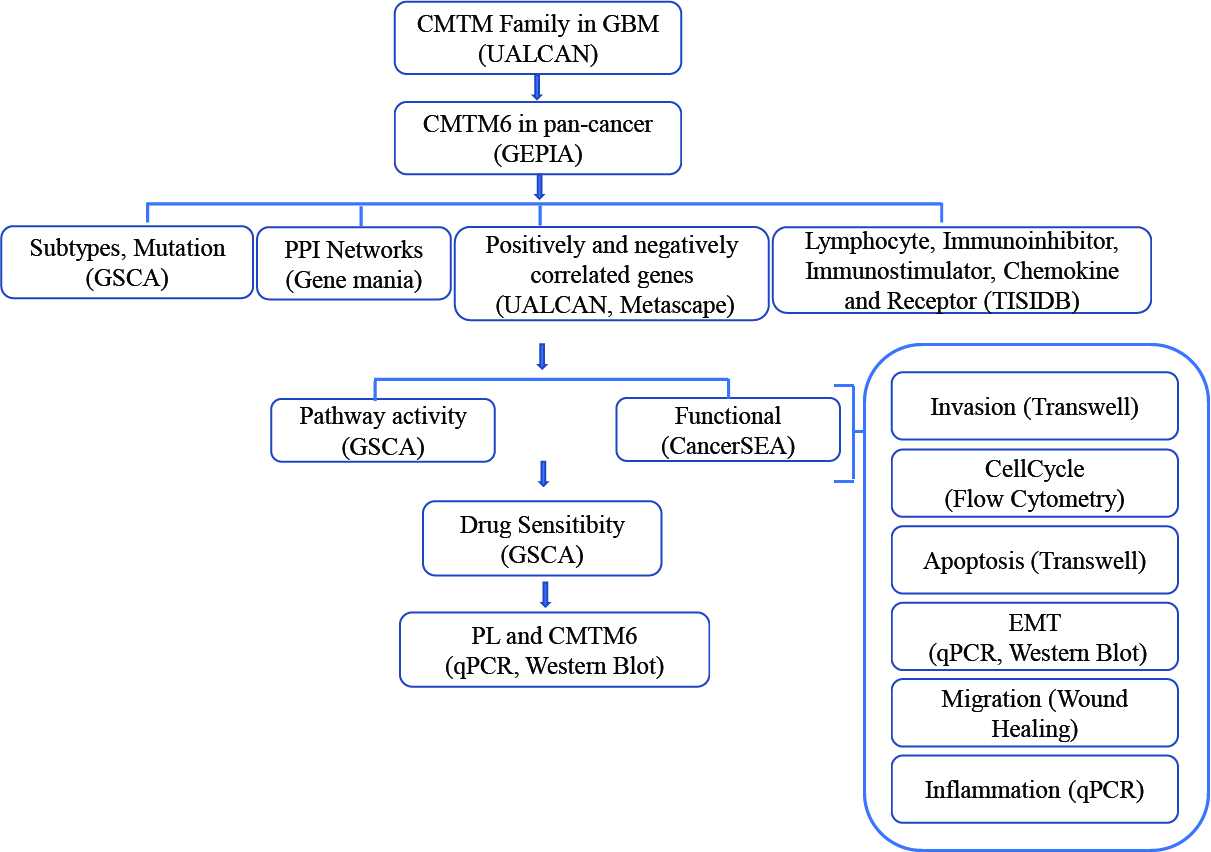

Supplement: Supplementary Figure S1 — Flow chart of this study. [file Image_1.tif]

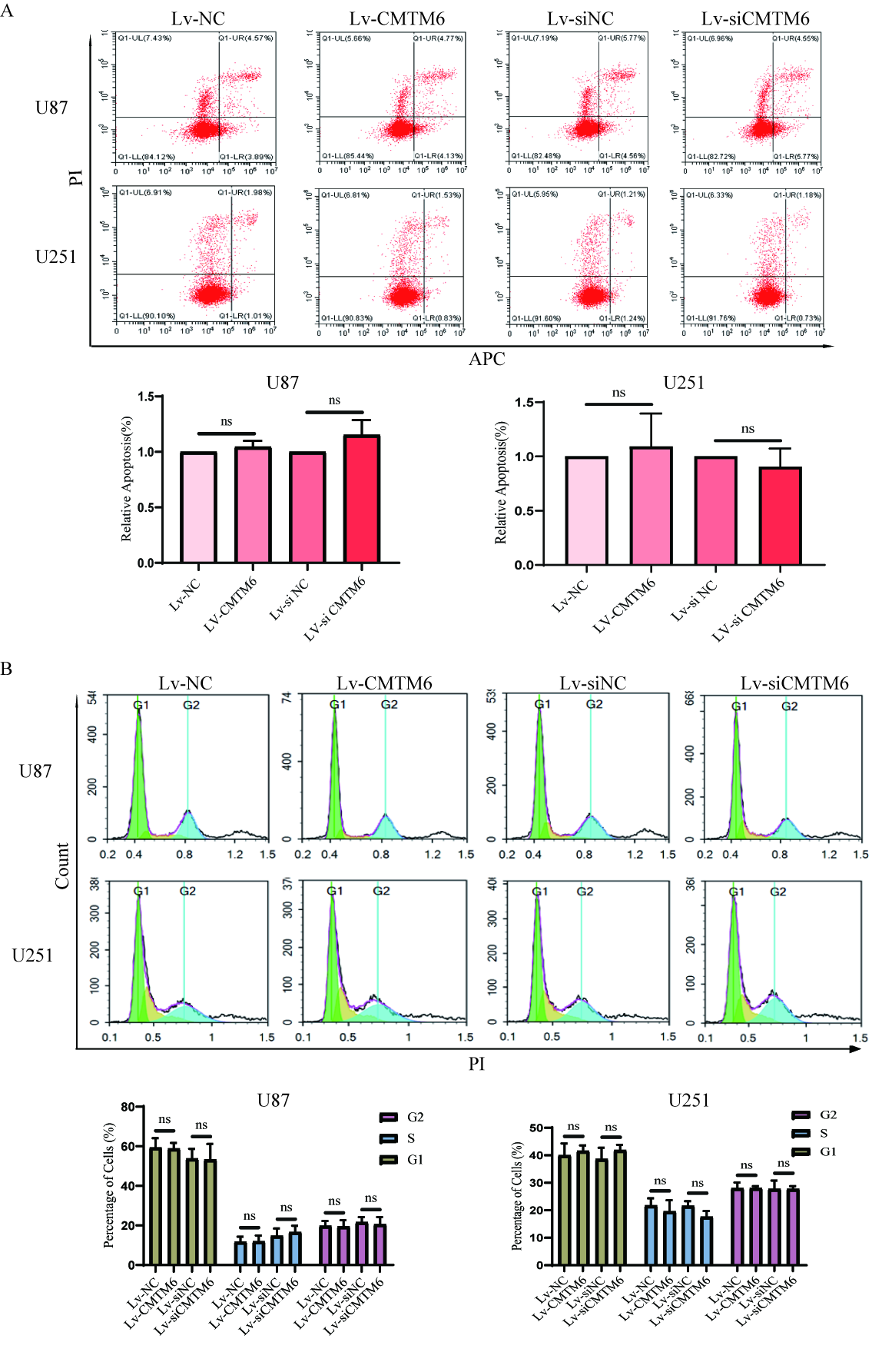

Supplement: Supplementary Figure S2 — The relationship between the expression of CMTM6 and the apoptosis and cellcycle of cells. (A) Flow cytometric apoptosis and result analysis of cells after up and down regulation of CMTM6. (B) Flow cellcycle and results analysis of cells after up and down regulation of CMTM6. *p<0.05. [file Image_2.tif]

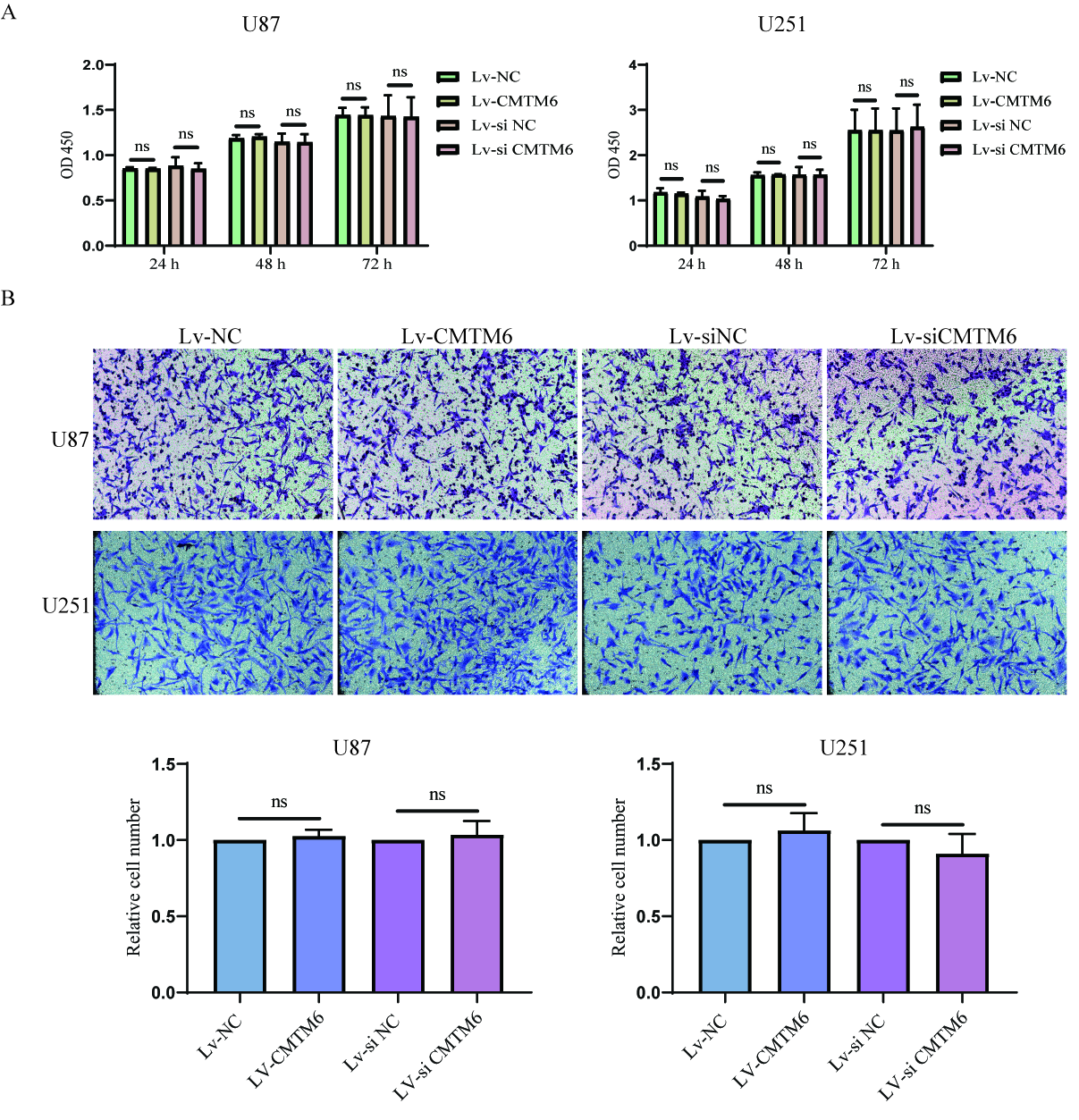

Supplement: Supplementary Figure S3 — The relationship between the expression of CMTM6 and the proliferation and invasion of cells. (A) Changes in cell proliferation ability at 24 h, 48 h and 72 h after up and down regulation of CMTM6. (B) Transwell invasion and result analysis of cells after up and down regulation of CMTM6. *p<0.05. [file Image_3.tif]
