# Supplementary material for: Chemokine-like factor-like MARVEL transmembrane domain containing 6: Bioinformatics and experiments in vitro analyze in glioblastoma multiforme
Source: Front Mol Neurosci. 2023 Jan 9;15:1026927. doi: 10.3389/fnmol.2022.1026927 (PMC9869805; doi:10.3389/fnmol.2022.1026927)
Supplement: Supplementary file 4 [file Table_1.docx]

| Correlation with CMTM6 | Chemokine | Receptor | | *P* value |
| --- | --- | --- | --- | --- |
| Positive correlation | CCL3. CCL4, CCL19, CXCL1, CXCL2, CXCL11, CXCL12, CXCL14 | CXCR1, CX3CR1 | *P*＜0.05 | |
|  | CCL2, CCL5, CCL7, CCL8, CCL13, CCL14, CCL18, CCL20, CCL22, CCL26, CXCL3, CXCL5, CXCL6, CXCL8, CXCL9, CXCL10, CXCL13, CXCL16 | CCR1, CCR2, CCR5, CCR7, CXCR2, CXCR3, CXCR4, CXCR6 | *P*＜0.0001 | |
| Negative correlation | CX3CL1 |  | *P*＜0.05 | |
| No correlation | CCL28 | CCR6, CCR10 | *P*＞0.05 | |
| No expression | CCL1, CCL11, CCL15, CCL16, CCL17, CCL21, CCL23, CCL24, CCL25, CCL27, CXCL17, XCL1, XCL2 | CCR3, CCR4, CCR8, CCR9, CXCR5, XCR1 |  | |

Table S1 Correlation of chemokine and chemokine receptor with CMTM6 expression.
