# Supplementary material for: Chemokine-like factor-like MARVEL transmembrane domain containing 6: Bioinformatics and experiments in vitro analyze in glioblastoma multiforme
Source: Front Mol Neurosci. 2023 Jan 9;15:1026927. doi: 10.3389/fnmol.2022.1026927 (PMC9869805; doi:10.3389/fnmol.2022.1026927)
Supplement: Supplementary file 5 [file Data_Sheet_1.docx]

**Fig 6B** Western blot results of CMTM6 expression after up and down regulation of CMTM6.

U87

U251

Lv-NC Lv-CMTM6


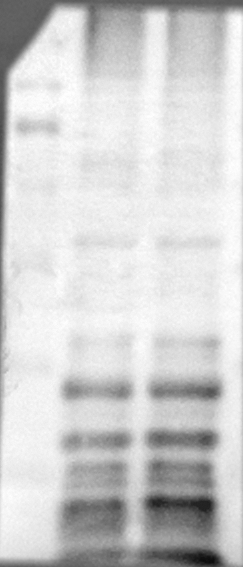

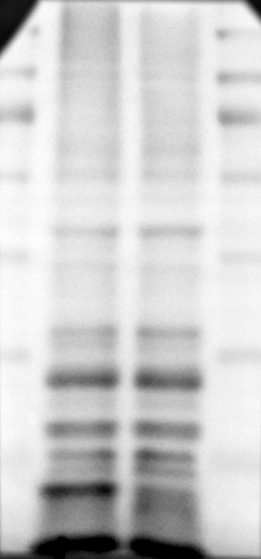

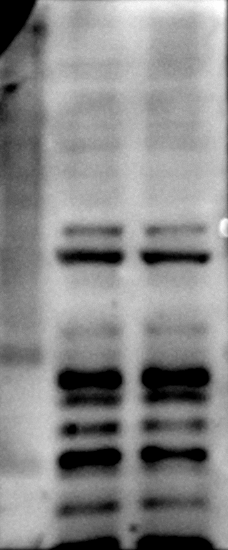

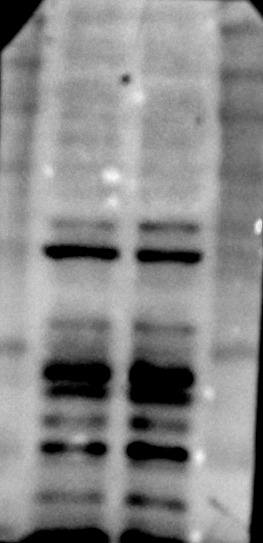

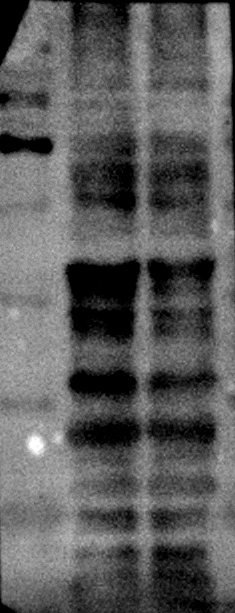

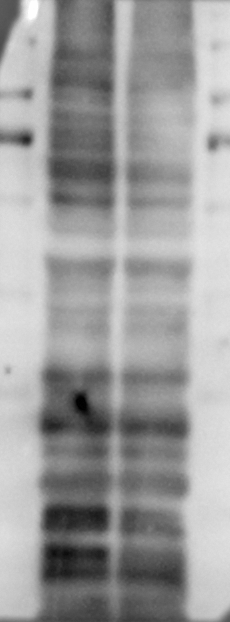

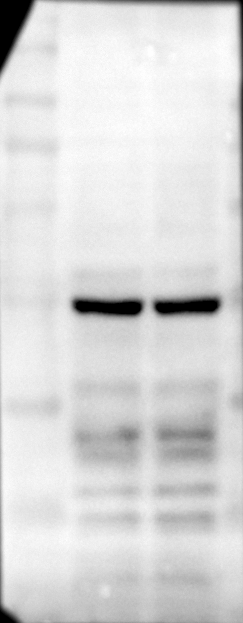

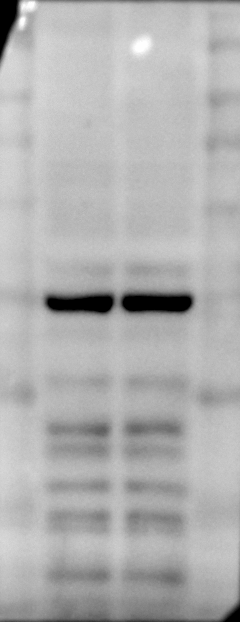


Lv-NC Lv-CMTM6

Lv-siNC Lv-siCMTM6

Lv-siNC Lv-siCMTM6

β-actin

CMTM6

22 kDa

43 kDa

**Fig 7B** Western blot results of N-Cadherin expression after up and down regulation of CMTM6.

U87

U251

Lv-NC Lv-CMTM6

Lv-NC Lv-CMTM6

Lv-siNC Lv-siCMTM6

Lv-siNC Lv-siCMTM6

β-actin

N-Cadherin

140 kDa

43 kDa


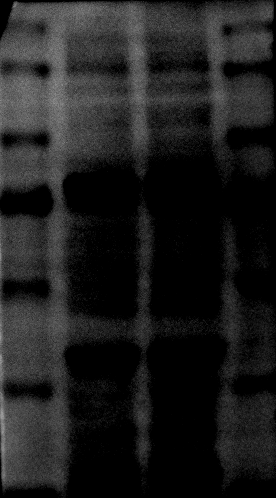

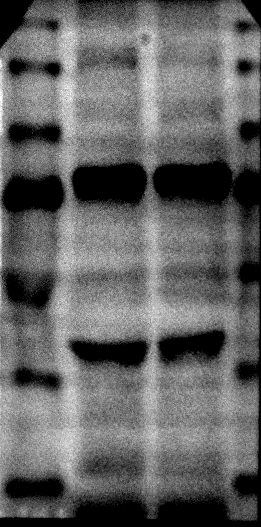

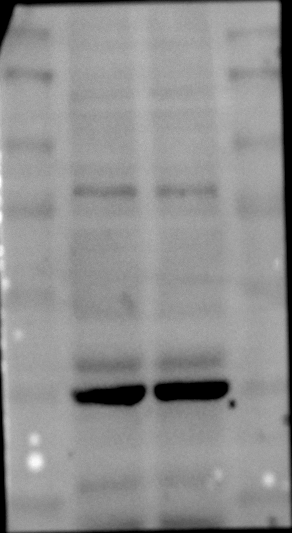

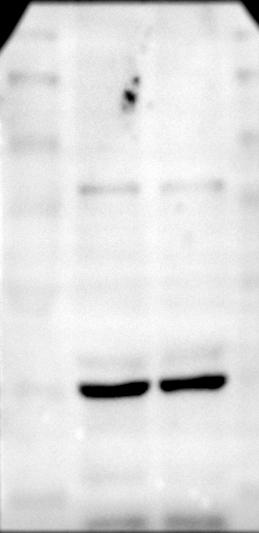

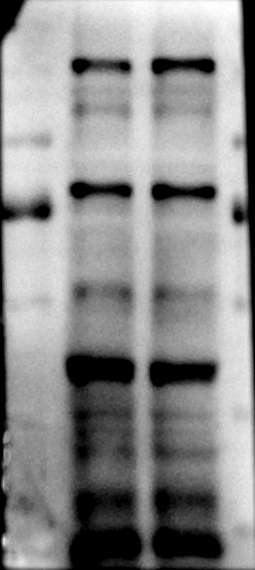

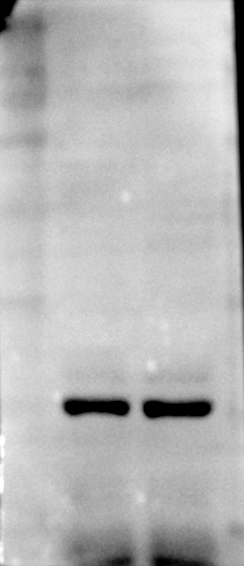

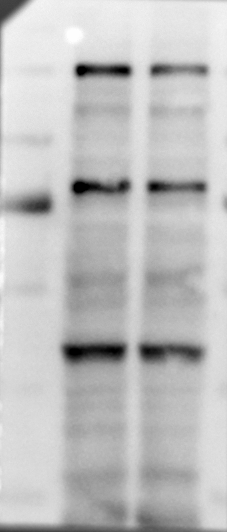

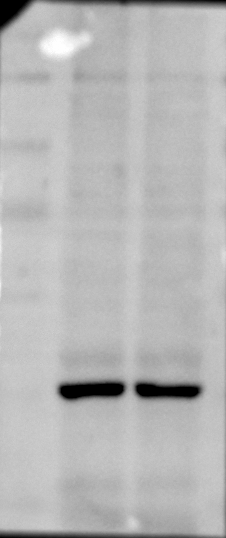


**Fig 7B** Western blot results of Vimentin expression after up and down regulation of CMTM6.

U87

U251

Lv-NC Lv-CMTM6

Lv-NC Lv-CMTM6

Lv-siNC Lv-siCMTM6

Lv-siNC Lv-siCMTM6

β-actin

Vimentin

57 kDa

43 kDa


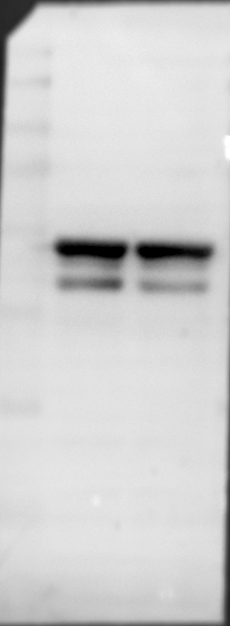

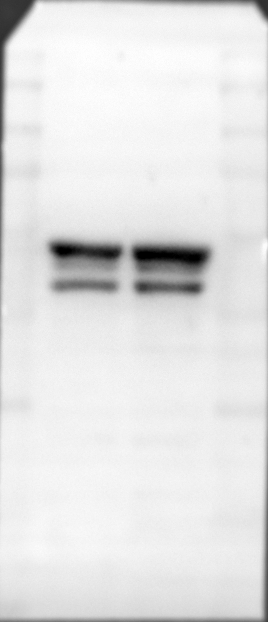

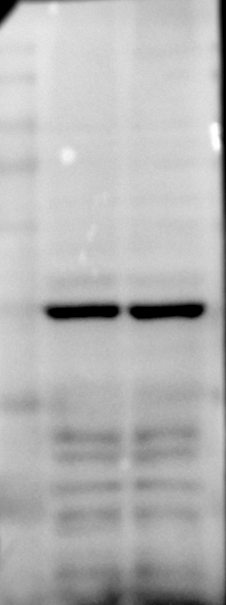

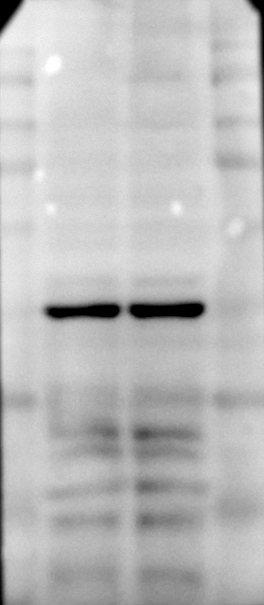

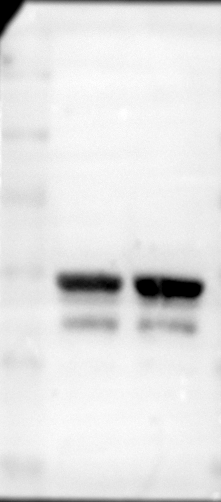

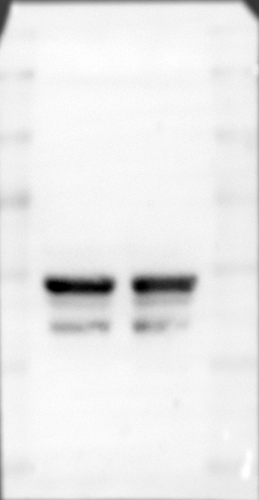

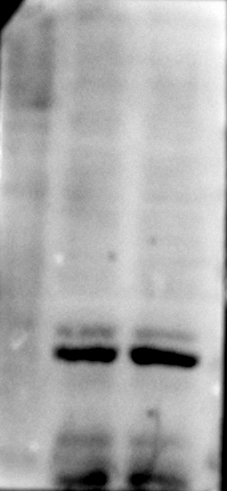

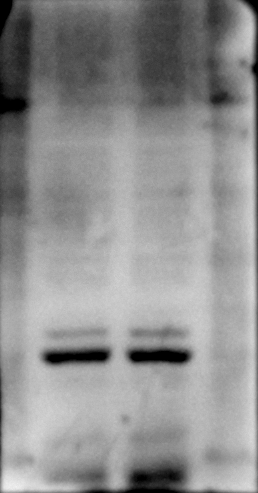


**Fig 9D** Western blot results of CMTM6 protein levels after 24 hours of PL action on U87 and U251 cells.

U87

U251

10 uM 0 uM

β-actin

CMTM6

22 kDa

43 kDa


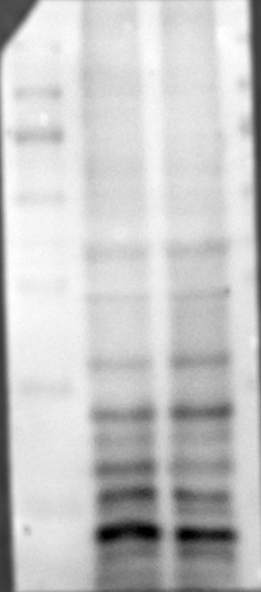

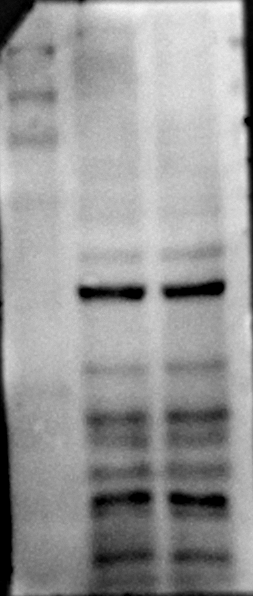

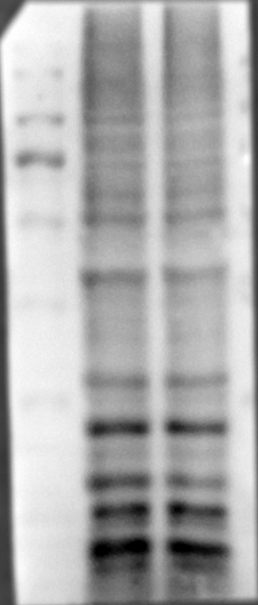

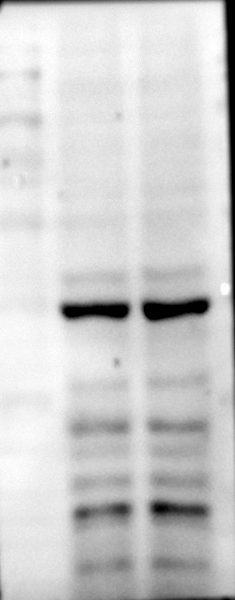


10 uM 0 uM
